# The efficiency paradox: A temporal lens into online dating among Chinese immigrants in Canada

**DOI:** 10.1177/02654075251339257

**Published:** 2025-04-28

**Authors:** Manlin Cai, Yue Qian, Yang Hu

**Affiliations:** 1 8166 University of British Columbia, Canada; 2 University College London, UK

**Keywords:** dating, immigration, intimate relationships, qualitative methods, technology

## Abstract

Online dating is widely assumed to enhance the overall efficiency of relationship formation through expanding the pool of potential partners. Yet little is known about how this presumed efficiency plays out beyond the initial search stage. Although temporal compression (i.e., saving time) is considered central to the notion of efficiency, individuals’ lived realities of time and efficiency in online dating remain understudied. Adopting a grounded theory approach to analyzing 31 in-depth interviews with heterosexual Chinese immigrant online daters in Canada, we reveal how time-related expectations and experiences shaped their perceptions of (in)efficiency throughout different stages of online dating. Specifically, our participants started with an efficiency expectation of temporal compression, expecting online dating to save time. As the dating process unfolded, however, they experienced inefficiency through diverse temporalities, including temporal suspension and simultaneity in mediated communication and temporal reconfiguration during modality switching. These experiences contradicted our participants’ initial efficiency expectation, prompting some to reevaluate their expectation and develop a preference for temporal slowdown in dating. Our findings highlight an “efficiency paradox” whereby the promise of efficiency not only runs counter to online daters’ lived realities but also amplifies perceptions of inefficiency. Foregrounding the voices of racial minority immigrants, our study challenges the commonly envisioned efficiency of online dating and provides new insights into how digital technologies mediate intimate lives through shaping individuals’ temporal experiences.

## Introduction

With rapid advancement of digital technologies, individuals increasingly use online dating to look for a romantic partner (Wu & Trottier, 2022). Dating sites and apps provide users with instant access to an expanded pool of potential partners and allow them to easily sift through a large number of profiles (Coduto & Fox, 2024). Given these attractive features at the initial search stage, online dating is often assumed to enhance the efficiency of relationship formation (e.g., Finkel et al., 2012; Rosenfeld, 2017; Rosenfeld & Thomas, 2012). Dating platforms also tout the promise of efficiency for users to find a partner and commonly cite the fast speed and high success rate of relationship formation as salient evidence of this efficiency (e.g., Bumble, 2025; eHarmony, 2024; Plenty of Fish, 2024; Tinder, 2025). The envisioned efficiency of online dating could be particularly appealing to racial minority immigrants, whose international relocation and minority status in the host society tend to limit their opportunities for meeting romantic partners through offline venues (Qian, 2022; Schwartz & Velotta, 2018).

While existing research has mostly attributed the efficiency of online dating to its role in expanding the pool of and facilitating access to potential partners (e.g., Finkel et al., 2012; Rosenfeld, 2017), the efficiency of online dating beyond the initial search stage remains underexplored. As a multistage process, online dating involves not only searching for a partner but also communicating online, transitioning to offline meetings, and eventually forming or failing to form a relationship (Sharabi, 2023). Despite increased dating opportunities, success in establishing a romantic relationship is far from a universal experience for online daters. Many users have never had in-person meetings with people they met on dating platforms (Timmermans & Courtois, 2018). Only 40% of online daters in Canada and the USA have ever entered a long-term relationship with someone they met online (Anderson et al., 2020; Qian, 2022). As we look beyond the initial search stage, the diverse experiences and divergent outcomes of “doing” dating online challenge a simplistic view on the efficiency of online dating.

Our study aims to complicate and rethink the concept of efficiency in online dating by adopting a temporal lens. A temporal lens entails considering how individuals’ time-related expectations and experiences, as well as the discrepancy between the two, shape their views on (in)efficiency throughout the online dating process. Navigating a process inevitably entails experiencing the passage of time; efficiency, as it is commonly understood, involves saving time (Adam, 2013). Digital technologies are widely believed to speed up processes of transportation, production, and communication, and scholars have conceptualized the experience of speeding up as temporal compression (Harvey, 1989; Rosa, 2003). Dating technologies are no exception. The perceived efficiency of online dating implies that it saves time for people to form a relationship (Finkel et al., 2012). Interrogating this assumption, we argue that online dating reconfigures individuals’ experiences of time in complex ways beyond mere temporal compression. Despite growing attention to people’s diverse temporal experiences in digitally mediated lives (e.g., Acedera & Yeoh, 2019; Lim, 2019; Liu, 2024), research has yet to examine such experiences in online dating and how they influence online daters’ efficiency perceptions.

Our study examines imagined and lived temporalities in online dating. In analyzing 31 in-depth interviews with heterosexual Chinese immigrants in Vancouver, Canada, we both compare across participants and trace each participant throughout the online dating process. Our findings reveal diverse temporalities within and across key stages of online dating, including temporal compression (expectation and initial search), temporal suspension and simultaneity (mediated communication), temporal reconfiguration (modality switching), and temporal slowdown (going offline). Our study centers on the voices of racial minority immigrants and points toward an “efficiency paradox” for minority groups, whereby the expected efficiency of online dating is particularly appealing to them but contradicts and amplifies their lived realities and perceptions of inefficiency in the online dating process. Our findings indicate that rather than following an expedited, linear progression from accessing a larger dating pool to forming a relationship, complex temporal experiences unfold throughout different stages of online dating. Our findings challenge the prevalent thinking that extrapolates an assumed efficiency in relationship formation from what online dating enables users to do at the search stage. We demonstrate the value of a temporal lens in advancing theoretical and empirical understanding of digitally mediated intimate lives.

## Efficiency in online dating: Beyond the search stage

Thanks to “its search technology and its presumably rich and extensive database to search from,” online dating enables individuals to meet potential partners whom they are unlikely to encounter offline (Rosenfeld, 2017, p. 492). Previous research often extrapolates the efficiency of online dating at the search stage into its role in enhancing the overall efficiency of relationship formation (e.g., Finkel et al., 2012; Qian, 2022; Rosenfeld, 2017; Rosenfeld & Thomas, 2012). This body of research also tends to infer online dating’s efficiency from the outcome that couples who met online have been on the rise—an approach that focuses on the “successful stories” while overlooking those who fail to form a relationship via online dating (Rosenfeld et al., 2019; Rosenfeld & Thomas, 2012).

Another line of research has argued that the abundant choices offered by online dating at the search stage do not necessarily lead to the efficiency of finding a partner because individuals may face greater difficulty committing to one single option (D’Angelo & Toma, 2017; Pronk & Denissen, 2020; Thomas et al., 2022). For example, Pronk and Denissen’s (2020) experimental study shows that when presented with more potential partners, individuals are more likely to reject dating candidates, are less likely to form romantic matches, and tend to perceive a lower level of dating success. Existing studies that challenge the efficiency of online dating have mostly been conducted in experimental settings rather than real-world contexts, and few of them have extended their explanations for the (in)efficiency of online dating beyond the search stage.

In this study, we highlight the imperative of examining how efficiency is understood and experienced by individual users as they navigate the entire process of online dating beyond just the search stage. Accessing an expanded pool of potential partners is only the beginning of online dating. Subsequently, users progress through multiple stages, including initiating contact, engaging in online mediated communication, and transitioning to offline meetings (Finkel et al., 2012). Users often move back and forth between these stages, and the process may or may not culminate in successful relationship formation. Going beyond evaluating efficiency based on aggregate rates of successful relationship formation, our study provides new insights into the efficiency of online dating as grounded in people’s lived realities from the bottom up.

## Efficiency in online dating: A temporal lens

What is efficiency? Social and economic theories typically conceptualize efficiency as a cost-return equilibrium: achieving maximum productivity (i.e., successful relationship formation) with minimum wasted effort or expense (Lefeber & Vietorisz, 2007). A crucial cost associated with dating is time, which remains understudied. As individuals navigate online dating, they inevitably experience the passage of time, and temporal perceptions (e.g., a waste of time) could powerfully shape how they evaluate the efficiency of online dating. To understand how efficiency unfolds, our study adopts a temporal lens that focuses on individuals’ time-related expectations and experiences throughout the online dating process.

Time is a fundamental organizing element of human life and has long been key to theorizing transformative changes associated with digital innovations (see Gotham, 2020 for a review). Scholars coined the concept of “temporal compression” to capture how technologies accelerated transportation, production, and communication, thereby enhancing efficiency by reducing the time required for various activities (e.g., Harvey, 1989; Rosa, 2003). Going beyond temporal compression, recent advancements in theorizing time have drawn attention to the multiplicity of temporalities—how different technological features create diverse temporal experiences and perceptions (Lohmeier et al., 2020; Wajcman, 2008). For instance, Lim’s (2019) study on parenting in the digital era showed that always-on mobile technologies expanded the temporal limits of parenting duties, which compelled parents to fulfill such duties ceaselessly in a “timeless time” (p. 5). Acedera and Yeoh’s (2019) study on digitally mediated communication among transnational couples found that the couples used synchronous calls to foster a shared “temporal rhythm” across time zones; they also used asynchronous messaging to create a “temporal rupture/suspension,” which allowed them to delay or withhold responses when they wished to avoid conflict. These examples suggest that the interplay between technological features and user agency creates diverse, dynamic temporal experiences in everyday life.

While a temporal lens has provided important insights into how digital technologies reconfigure various aspects of family life (e.g., parenting, communication; for a review, see Qian & Hu, 2024), it has yet to be used to understand how people experience (in)efficiency in online dating. Against this backdrop, below, we review existing research on the online dating process and discuss how our study advances this scholarship by foregrounding a temporal lens. We draw particular attention to people’s diverse temporal experiences throughout the online dating process as digital technologies relate differently to specific stages of online dating.

Initially, online dating platforms—with large user bases and advanced search functions—serve as a tool that helps users expand their dating pool (Rosenfeld & Thomas, 2012). After online daters gain unilateral access to an expanded pool of potential partners, the next key step is to establish bilateral contact, in which the role of digital technologies shifts toward mediating interactions between users (Finkel et al., 2012). Communication at this stage is typically asynchronous, which requires one person to initiate contact and then wait for the other to respond. Meanwhile, users can contact and communicate with multiple dating candidates simultaneously. Given the expectations for men to assume a proactive role and women a reactive role in heterosexual courtship (Lamont, 2020), existing research shows that men are generally more likely than women to initiate contact and send first messages (Kreager et al., 2014; Qian, 2022; Sharabi & Dykstra-DeVette, 2019). This encourages us to examine how temporal asynchronicity (waiting for responses) and simultaneity (communicating with multiple dating candidates concurrently) may lead to divergent experiences of time and efficiency for men and women in online dating.

After a period of online communication, daters typically seek to meet offline if mutual interest remains (Sharabi & Dykstra-DeVette, 2019). In this transition, digital technologies recede into a secondary role as online daters move from asynchronous, concurrent, and mediated communication, potentially with multiple candidates, to synchronous, one-on-one, in-person meetings. Here, research drawing on modality switching theory has focused on the timing of the online-to-offline transition (Ramirez et al., 2015; Ramirez & Zhang, 2007; Sharabi & Caughlin, 2017). Ramirez and colleagues (2015), for example, find that prolonged online interactions tend to negatively affect face-to-face meetings. This is because asynchronous text-based communication relies heavily on verbal cues and allows users ample time to craft responses (Ramirez & Zhang, 2007; Walther, 1996). As a result, prolonged online interactions tend to reinforce an idealized self-presentation, which often contradicts a fuller and less selective presentation revealed by diverse (nonverbal) cues in face-to-face meetings (Ramirez et al., 2015; Ramirez & Zhang, 2007). Although existing research has emphasized the timing of modality switching, insufficient attention has been paid to how the online-to-offline transition also entails temporal reconfiguration that can affect individuals’ understanding and experiences of efficiency in online dating.

## Empirical case: Chinese immigrants in Canada

The presumed efficiency of online dating is particularly appealing to minority groups, such as racial minority immigrants (Qian, 2022; Schwartz & Velotta, 2018). Given their experiences of diverse forms of marginalization and a tendency for racial endogamy, racial minorities often face a limited pool of potential partners in everyday offline life (Cai & Qian, 2023; Schwartz & Velotta, 2018; Williams, 2024). Adding to the challenge is immigrants’ transnational relocation, which often disrupts their preexisting social networks and makes it even more difficult for them to meet romantic partners through traditional offline venues, such as via the introduction of family or friends (Chen & Liu, 2021; Qian & Hu, 2025). Despite online dating’s strong appeal to racial minority immigrants, the question remains as to how they experience efficiency in online dating.

To answer this question, we focus on Chinese immigrant online daters in Vancouver, Canada, as a salient case. As of 2022, 94% of the Canadian population had internet access (Denier et al., 2024). With nearly ubiquitous internet use in Canada, online dating has gained popularity: The number of users increased from 1.8 million in 2017 to 2.9 million in 2023 (Statista, 2024). In addition, according to the 2021 Census, immigrants accounted for about a quarter of Canada’s population. Vancouver is one of the largest immigrant-receiving metropolitan areas, where 47% of residents were immigrants in 2021 (Statistics Canada, 2022). Chinese immigrants accounted for 32% of Vancouver’s immigrant population in 2021, representing the city’s largest racial minority immigrant group (Statistics Canada, 2022). By focusing on the lived experiences of Chinese immigrant online daters in Vancouver, our study reflects critically on popular discourses that cast online dating as an efficient solution to “dating problems” faced by individuals with (intersecting) minority identities (EME Hive, 2024; Schwartz & Velotta, 2018). We expect our conceptual insights to be relevant to other minority groups in a broader range of contexts beyond Chinese immigrants in Vancouver.

## Methods

### Sample

This study is part of a bigger project examining heterosexual online dating. We draw on in-depth interviews with 31 Chinese immigrants who have used online dating in Vancouver, Canada. We mobilized diverse channels to recruit participants. We distributed recruitment flyers through community centers, coffee shops, and gyms across Vancouver and posted digital advertisements via forums such as Reddit, social media, local Chinese-language online newspapers, and classified advertisement websites tailored to the Chinese community in Vancouver (ethnic versions of Craigslist). Personal connections and snowball sampling were also used for recruitment.

Our sample consists of 17 cisgender women and 14 cisgender men, with a mean age of 33 years. About 45% of our participants were in their 20s, 39% were in their 30s, and 16% were 40 or older. All but one had a bachelor’s degree or above, which partly reflects the positive socioeconomic selection of immigrants into Canada (Schinnerl & Ellermann, 2023). Twelve participants arrived in Canada before the age of eighteen, and nineteen immigrated as adults. Most participants tried multiple dating sites/apps but typically used one primary platform, including Tinder, Coffee Meets Bagel, Plenty of Fish, and Chinese-oriented platforms such as Two Red Beans and Tantan. Regarding relationship goals, eighteen explicitly looked for a serious relationship (e.g., marriage or a long-term relationship), whereas only four mainly looked for casual encounters and hookups. The other nine were open to different possibilities without a specific goal, which reflects the ambiguous and flexible nature of partner search, as even casual encounters could evolve into serious relationships (Timmermans & Courtois, 2018). Although our participants used a variety of dating platforms and had varied relationship goals, expectations and diverse experiences pertaining to time and (in)efficiency featured prominently in their online dating process.

### Data collection and analysis

In 2018 and 2019, the second author (the project Principal Investigator [PI]) and a research assistant conducted in-person semi-structured interviews. This study received ethics approval from the PI’s institution. With informed consent from all participants, the interviews were audio-recorded and transcribed verbatim. The interviews ranged between one and three hours, with a mean of approximately two hours. Interview topics included participants’ sociodemographic background, past relationships, reasons for using online dating, partner preferences, platform choices, experiences across various online dating stages, and views on dating and marriage. Twenty-six participants were interviewed in Mandarin, and the other five were in English. In writing this article, we translated the Chinese quotes into English, and all authors cross-checked the translations to ensure accuracy. Pseudonyms were used to protect participants’ anonymity.

Informed by theories and empirical insights from the ground up, our data analysis involved multiple rounds of iterative coding. During initial line-by-line coding (Glaser & Strauss, 2017), the first author read the transcripts and disaggregated the data into subsections with summary codes. This process revealed shared expressions from participants capturing time-related concerns, such as “lots of work/labor,” “a waste of time,” and “time-consuming,” with some participants explicitly using the term “inefficient” to describe their online dating experience. All authors reviewed relevant codes and quotes; after in-depth discussions, we identified (in)efficiency, especially concerning time, as a salient overarching concept cutting across participants’ motivations for and experiences of using online dating. Next, the first author conducted focused coding (Charmaz, 2014) to identify subthemes related to (in)efficiency. The second and third authors independently reviewed the codes and corresponding quotes. This cross-checking process focused not only on verifying the codes but also, more importantly, on how to make sense of the discursive data. Following the tradition of a grounded theory approach (Glaser & Strauss, 2017), all authors applied their theoretical expertise and contextual understanding to deliberating their interpretations of the qualitative data before reaching a consensus. Through iterative analyses and deliberations, we identified our participants’ temporal expectations and experiences at different online dating stages as key subthemes. Finally, building on theories and prior research on temporalities, we conducted axial coding to synthesize the specific codes related to time and (in)efficiency into a coherent conceptual framework (Strauss & Corbin, 1997). The axial coding process involved (1) organizing codes on temporality and (in)efficiency by key online dating stages, (2) within-case analysis to understand changing temporalities across online dating stages, and (3) cross-case comparison to capture participants’ shared and divergent experiences at each stage.

## Findings

In this section, we elaborate on our participants’ expectations and experiences of time and (in)efficiency throughout key online dating stages. We first show what the participants expected from online dating when they opted for it. Then, we discuss their temporal experiences and the implications of these experiences for (in)efficiency at the stage of mediated communication (i.e., online messaging). Next, we consider temporal experiences and the (in)efficiency therein in the online-to-offline transition. Finally, we examine how some participants reevaluated their efficiency expectations in online dating, decided to take their time in dating, and developed a preference for seeking a partner through offline venues. Because gender differences in the online messaging stage are well documented in prior research (Kreager et al., 2014; Qian, 2022; Sharabi & Dykstra-DeVette, 2019) and are also prominent in our data, we present the findings for men and women separately for this stage. However, we have not observed systematic gender differences pertaining to temporality and (in)efficiency at the other stages.

### The promise of efficiency in online dating

As vividly exemplified by the name of the dating app “Plenty of Fish,” online dating is widely believed to enhance the efficiency of finding a partner because it expands the pool of potential partners at the search stage (e.g., Finkel et al., 2012; Qian, 2022; Rosenfeld, 2017; Rosenfeld & Thomas, 2012). This common understanding of efficiency echoed our participants’ expectation that online dating would allow them to meet more people easily. Meanwhile, our participants expressed an equally prominent expectation of *temporal compression*, whereby online dating would require less time investment compared to traditional offline dating. In this section, we examine our participants’ efficiency expectations as they embarked on the journey of online dating.

“Who do you think uses online dating?” When asked this question, Ligang (man, 27), who immigrated to Canada at the age of 21 and worked as a busy professional after university, answered: “Those with smaller social circles and limited time.” As Ligang’s answer succinctly captured it, the promise of efficiency in online dating through expanding the dating pool and overcoming temporal constraints was particularly appealing to our immigrant participants. More than a third of our participants mentioned restricted social circles as a formidable barrier in their partner search offline. Because of small social circles, Chuyi (woman, 50) felt that she had “no choice but to rely on online dating,” a sentiment echoed by participants like Jiajing (woman, 31). Liuzhao (man, 32), who migrated to Canada at age 23, preferred Chinese immigrant women as potential partners but faced the challenge of meeting them in offline settings, such as at work: “None of my colleagues are Chinese, and they are all men. So, my dating prospects look grim.” Similarly, Zhilin (woman, 33), who moved to Canada at age 24, recalled that more than four years after her arrival, she still had few friends: “It’s difficult to expand [social circles], be it making friends or finding a partner. In social life, the beginning is tough for newcomers like us who didn’t grow up here.” Zhilin tried socializing through in-person activities where new immigrants gathered to practice English, but found that people “didn’t want to chat and went home right after class.” After several unfruitful attempts, Zhilin realized that it was “so difficult to meet someone” through friends, work, or other offline venues, which prompted her to use online dating.

Besides a small dating pool, “there’s little time left to actually socialize,” as Daisy (woman, 37) complained. Due to language and cultural barriers, our immigrant participants felt that study and work could be all-consuming, leaving them with little time to look for a partner offline. In this context, many participants, like Meimei (woman, 29), expected online dating to be time-saving: “Why do people enjoy dating apps? It’s the same reason people like TikTok—because it’s fast. People enjoy efficiency.” The promise of efficiency through temporal compression attracted many of our participants to use online dating. Xiaoqian (woman, 28), who first immigrated to Canada for college, explained, “English was my second language. In psychology, we had many writing- and reading-intensive courses, so I spent lots of time studying rather than socializing.” She began to use online dating because she “wanted to quickly build up” a relationship. Weimin (man, 31), who had lived in Canada for seven years and was a warehouse assistant manager at the time of the interview, devoted most of his time to work. Weimin found it time-consuming to meet women through social activities such as karaoke and group dinners, and he went for online dating “precisely because [he] didn’t have time.” Richard (man, 25) similarly expected online dating to be less laborious and time-saving: “Meeting new people [in person] is a chore, whereas, for online dating, you don’t have to do anything. You just swipe.”

Did online dating fulfill its promised efficiency? Our participants recognized that online dating did expand their dating pool. Ligang (man, 27), who described himself as a homebody (*zhainan*), recounted what he liked most about online dating: “You can do it all from home. With other ways, you at least have to meet in person to make new friends.” Kaiwen (woman, 48) liked online dating because it allowed her to meet people outside of her friendship and social circles. Similarly, Duanli (woman, 37) considered expanding the dating pool to be the greatest benefit of online dating:

In traditional ways of meeting partners, no matter how, you meet those in proximity to you, like your coworkers, friends of coworkers, neighbors, or schoolmates, who are all in your circle. When it comes to some people I met online, if not for the internet, I would have never encountered them in my lifetime…They don’t overlap with my circle at all. This counts as the biggest advantage [of online dating].

Our findings in this section show that the experiences of dating pool expansion at the search stage largely aligned with our participants’ efficiency expectations. However, their subsequent experiences of online dating departed from the expected temporal compression. Next, we turn to how their experiences and perceptions of (in)efficiency unfolded through complex temporalities beyond the initial search stage.

### Temporalities and (in)efficiency in mediated communication

While online dating provides users with unilateral access to a large pool of potential partners, a key step toward relationship formation is establishing bilateral communication through conversations with interested candidates (Finkel et al., 2012; Sharabi & Dykstra-DeVette, 2019). Despite diverse modes of digital communication, our participants relied primarily on messaging. Because messaging is asynchronous and does not require immediate responses, it can create *temporal suspension* (for those waiting for a reply) and *temporal simultaneity* (as communication with multiple people at the same time is made possible). The dominant gender dynamics of men initiating and women responding in heterosexual dating (Lamont, 2020) created distinct temporal experiences at the messaging stage for the men and women in our study, leading both genders to question the efficiency of online dating.

#### Initiating contact: Temporal suspension for men

Among our participants, all but two men initiated contact, whereas women typically waited for messages and selectively replied (except on the dating app Bumble where women must message first). This gendered pattern is consistent with findings from previous studies (e.g., Kreager et al., 2014; Qian, 2022; Sharabi & Dykstra-DeVette, 2019). Going beyond existing research, we reveal how the gendered politics of mediated communication shaped our participants’ temporal experiences and contributed to their perceived inefficiency of online dating.

The men in our study frequently initiated contact. What then followed was often a prolonged abeyance of waiting for a reply that never arrived. This waiting game often left the men feeling uncertain about whether and when they would receive a reply, which created a sense of *temporal suspension*. Many estimated receiving a response from only 5–10% of the women they messaged. As Shouke (man, 31) described, “It’s quite difficult because I sent dozens of messages but didn’t get a single reply.” Similarly, Yuhan (man, 29) lamented: “The majority of women gave me the cold shoulder.”

The lack of success in getting a response, as experienced by men like Shouke and Yuhan, often drove them to cast a wider net by messaging a large number of women, which in turn magnified the potential suspense of waiting for a reply. Even when contact was established, the communication often fizzled out. Haoxiang (man, 43) explained why he thought men repeatedly encountered setbacks at the messaging stage:

This is about guys making the first move…When he finally gets a match, he has to find ways to keep the conversation going. A guy might be talking to three women at once, but a woman can be chatting with ten guys at the same time. Sometimes, at first, she talks to you, and you think things are going well, but she’s just available at that moment. A little later, she may start talking to five other guys, and if two of them catch her attention, she stops talking to you.

The uncertainty surrounding receiving a reply and sustaining communication kept the men in our study in constant suspense. This experience of temporal suspension, coupled with low response rates, took a toll on many men in our study, making them feel “constantly ignored,” “frustrated,” “disheartened,” “hurt,” and doubtful about whether they were “really that bad.” Such frustration led them to question the promise of efficiency with which they started online dating. Haoxiang, like many others, rejected the idea of efficiency: “The return doesn’t match the effort put in.”

#### Subsequent communication: Temporal simultaneity for women

Did the abundant messages received by women make online dating more efficient for them? In our study, women’s experience of mediated communication in online dating was characterized by *temporal simultaneity*. For many of the women, their dating site/app inboxes were flooded with messages awaiting their response. Thus, they had to tediously sift through numerous messages and juggle conversations with multiple potential partners. Michelle (woman, 26) shared her feeling of being overwhelmed: “I’m talking to two people I met on Tinder, plus there’s one other guy who I have rescheduled seeing. Because I’ve been busy, I just feel that’s too much already. There’s a lot of messaging.”

Dividing their already limited time between multiple candidates often made our women participants feel so overloaded that they struggled to focus on any one person, which diminished the efficiency of online communication in achieving the goal of establishing a meaningful connection and finding a suitable partner. Gaoyun (woman, 27) explained the difficulty of “[engaging] in deep conversations” on dating platforms:

Everyone is overwhelmed with so much information and so many strangers. It’s difficult to focus. Even if I wanted to talk to just one guy this week, it’s almost impossible, as the app keeps pushing new [matches and messages] to you non-stop…I don’t have the energy, nor would any other users, to get to know and chat with strangers every day.

Duanli (woman, 37) also grew weary of monotonous and repetitive conversations in online dating that seemed a waste of time to her:

You keep meeting new people, and each time you meet someone, you have to tell your story all over again. After doing it hundreds of times, it gets exhausting. Like when someone asks what you do for work, you think, “oh, here we go again,” and start explaining your job.

Like many other women in our study, both Gaoyun and Duanli realized that conversing with multiple strangers online demanded time and usually led to superficial connections.

Existing research shows that online daters often find their text-based exchanges on dating apps shallow and boring because text transmits a narrow range of cues and only allows for limited interactivity (Coduto & Fox, 2024; Suenzo, 2024). Extending these insights, we find that the experience of temporal simultaneity made it notably difficult for our women participants to invest time in cultivating deep connections. Meanwhile, online dating amplified the number of potential partners these women got in touch and conversed with, which intensified their temporal expectation for establishing an instant rapport through expedited communication and fostered a perception of potential partners as disposable. Meimei (woman, 29) illustrated this point:

Because dating apps are so fast-paced, either you immediately hit it off and have a great conversation, or things progress slowly, and before you reach the point to really connect, you cut them off because you’re overwhelmed by messages from others…And since it’s a fast platform, missing out on someone doesn’t matter because there are plenty of others waiting. The cost of missing out is so low—after all, I didn’t know you beforehand, and I don’t really know what you’re like. Would I feel it’s a pity if I miss out on you? No.

These observations led Meimei to conclude: “On dating platforms, it’s extremely rare to meet someone with whom you can truly connect.” Meimei was not alone. The fast yet engaging communication expected by women often fell through in their experiences of messaging multiple potential partners simultaneously. Some women in our study came to realize that in terms of establishing deep and meaningful connections, online dating hardly made things more efficient.

### Temporal reconfiguration and (in)efficiency in modality switching

Most of our participants ultimately sought to form a relationship offline. Thus, after establishing contact and engaging in conversations online, a crucial turning point would be making the transition to an in-person meeting. This transition has been the subject of research on modality switching, which has focused on when people make this transition in the online dating process and its consequences for subsequent relationship development (Ramirez et al., 2015; Ramirez & Zhang, 2007; Sharabi & Caughlin, 2017). Extending prior research, we draw attention to modality switching as a reconfiguration of temporalities in dating.

As shown in the preceding sections, our participants’ efficiency expectations often clashed with their experiences of inefficient online communication. This disconnect prompted many participants to speed up the transition to offline meetings, typically within a week of initial contact. This urge for modality switching stemmed primarily from a sense of time pressure and a desire to expedite the dating process. For instance, Gaoyun (woman, 27), whom we met in the previous section, considered online communication with strangers to be time-consuming. As a result, she preferred to meet face-to-face “not very long” after the start of online exchanges, usually “after two or three chats.” She explained that “I don’t have time to keep chatting on my phone. It’s not a good use of time…Everyone has work or other things to do. People aren’t very responsive. I’m not very responsive either.” Similarly, Fanghua (woman, 34), a nutritionist with a hectic work schedule, remarked: “I don’t have time. I feel [online chatting] is really slow.”

Although modality switching was often prompted by a pursuit of efficiency, did it actually save time? Not quite, given the drastic change of temporalities involved in this process. The online-to-offline transition entailed temporal reconfiguration from asynchronicity (messaging at any time) and simultaneity (multiple contacts and conversations) to synchronicity and exclusivity. Each face-to-face meeting constituted a separate, synchronous, and exclusive activity with one potential partner that required our participants to schedule a date and coordinate a place—tasks that demanded additional time and effort beyond those required at the messaging stage. As Duanli (woman, 37) described, “On the day I meet someone, I have to clear my schedule and make time specifically to meet him. Since he’s a stranger, I need to put in a lot of effort and concentration to interact.” Yangli (woman, 32) enumerated the time cost of meeting someone for lunch: “If you meet in person for lunch, it’ll take at least an hour. Going to a restaurant involves chatting, ordering food, waiting for it, eating, and then chatting some more—it could easily take one or two hours.” Gaoyun (woman, 27) further explained why going to an offline meeting took up more of her time: “It can take more than one hour just to travel to and from a meetup. As a woman, I also need to spend time on makeup and dressing up.”

Making time is, admittedly, a routine part of dating. Yet, why did our participants perceive modality switching to be such a time-consuming and inefficient process? Here, it is important to revisit our participants’ initial temporal expectations. When the participants first started using online dating, they expected efficiency through temporal compression. Recall that Meimei (woman, 29) remarked, “Why do people enjoy dating apps?…because it’s fast,” and Richard (man, 25) noted, “You don’t have to do anything. You just swipe.” At the modality switching stage, however, our participants’ lived experiences increasingly departed from their efficiency expectations as they invested a nontrivial amount of time in coordinating the online-to-offline transition and meeting face-to-face one-on-one with an expanded pool of potential partners. It is thus crucial to recognize that our participants’ initial efficiency expectations had a cascading impact on their temporal experiences and efficiency perceptions at subsequent stages of online dating.

While Duanli mentioned earlier that meeting with one person offline was already time-consuming and inefficient, engaging in one-on-one meetings with multiple candidates often led to a perceived temporal “explosion” and mental exhaustion. Gaoyun shared her experience:

Things just exploded. For example, between Monday and Friday, there were four to six guys asking me out this weekend. I was totally drained. They asked what my plan was for the weekend and suggested taking me out to do something. One guy offered a variety of fancy activities for me to choose from, but I told him I was feeling a bit tired.

Similarly, Chenhua (man, 30) felt exhausted:

Actually, it’s quite exhausting at times. You don’t know what they’re really like, so you feel like you have to give everyone the same time and effort…If three people replied to your messages, then you need to meet three people around the same time. Plus, everyone has their own friends and work to deal with. This is why I feel very tired.

Like Gaoyun and Chenhua, our participants often found making the transition to meet multiple dating candidates offline to be tiring and time-consuming rather than efficient.

Despite time coordination and investment, most offline meetings turned out to be unfruitful, which further reinforced our participants’ perception of wasted time and inefficiency in modality switching. Kate (woman, 39) met four men in person through online dating, but “nothing happened.” Duanli (woman, 37), who “[needed] to put in a lot of effort and concentration to interact [with a stranger],” met more than ten men face-to-face, and over half of these interactions concluded after the first meeting. When evaluating her experiences of these offline meetings, Duanli highlighted the significance of time investment: “For every meetup, paying for coffee and such is just a small expense. What matters to me is the cost of time.” Contrary to our participants’ expectations of temporal compression, modality switching entailed temporal reconfiguration, demanded substantial time investment, and was often perceived as inefficient.

### Going offline: Temporal slowdown and reevaluating efficiency

The accounts from the previous sections clearly indicate that contrary to their expectations, most of our participants did not experience the efficiency associated with temporal compression in online dating. Reflecting on their experiences, some considered the notion of efficiency an illusion. Zhaibo (man, 57) put it bluntly: “In online dating, you seem to meet lots of people, but in reality, I think the efficiency is very low.” Sam (man, 25) found his online dating experience “disappointing” and did not believe online dating was “actually more time-saving” given the limited quality time for meaningful interactions that he had gained from using dating apps. Similarly, when explaining why she stopped using Tantan (a dating app), Bingxian (woman, 27) said: “I realized that the time spent on it wasn’t worth it.” Time also featured prominently in Qingya’s (woman, 29) cost-benefit reflection on online dating: “Dating apps are a way to meet more people, but…the return on investment is unclear. Although it looks like there isn’t much investment, we’ve spent time on it.” For Xiaoqian (woman, 28), online dating was “a waste of time” considering its low success rate: “It’s like cold calling. If you make 100 calls and get one suitable person, that’s already pretty good.” Online dating also turned out to be “not of much use” to Mingqi (man, 47), whose feelings changed from initial excitement to eventual fatigue:

I was really excited at first, like a door had opened for me. You paid for the membership and suddenly you could meet so many women...So many opportunities. But then I realized I couldn’t even manage to set up any meetups, or when I ever did, the women I met weren’t what I expected. It just became really exhausting.

Looking back, many participants felt that the online dating process was drawn out. Comparing online and offline ways of meeting potential partners led them to reevaluate their efficiency expectations that online dating would help speed up the process of finding a partner and save time. When asked whether she would continue using online dating, Xiaoqian said she would keep it as an option but lower her expectations: “At first, I had unrealistic fantasies about quickly finding a suitable partner. Then I became disappointed, but I’ve adjusted my expectations. I’ve shifted from directly looking for a romantic partner to simply meeting someone interesting. Take it easy.”

Departing from a preoccupation with fast-paced, efficiency-oriented dating, some participants expressed a preference for returning to offline venues to seek a partner. At the time of the interview, Xiaoqian was dating a man introduced by her friend after she had used three dating apps intensively for three months and met about fifteen men without progressing further with anyone. She described her approach to cultivating her current relationship:

A good friend introduced us. I’m more willing to invest time and energy. By contrast, for someone [online] who just appears out of nowhere, I’d be more likely to give up at the first sign of discomfort, thinking, “Why should I keep meeting him?”

Xiaoqian’s reflections suggest that, in contrast to what she had looked for in online dating, the pursuit of efficiency was no longer her priority. Moreover, she expressed a greater willingness to invest in potential partners with whom she had offline connections compared to someone she met online without prior ties. Although Mike (man, 21) went out with around 30–50 women over two and a half years of online dating, he “moved on to meeting people in real life” to “get to know them better.” Similarly, Mingqi came to view online dating as a way of expanding his social network rather than an efficient way of forming a romantic relationship. Compared with online dating, he preferred offline ways of meeting, where people would “take time to get to know and understand each other.”

After a drawn-out, taxing online dating process, many of our participants began to challenge the very idea of efficiency in online dating. As our participants reconsidered their efficiency expectations, many became more willing to take their time in finding a partner and developed a preference for meeting potential partners through offline venues. Such reflections and preference for going offline reveal that efficiency expectations often paradoxically exacerbated the experiences of inefficiency and provoked subsequent frustration in online dating.

## Discussion and conclusion

**Figure 1.**
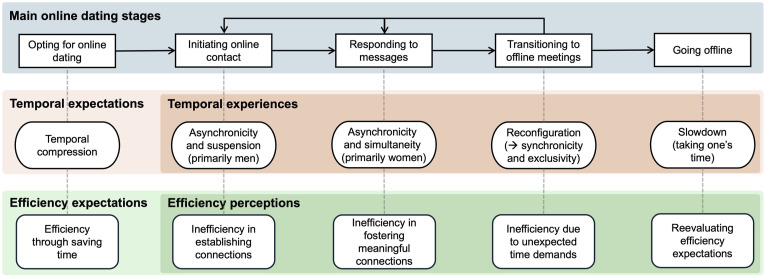
Temporalities and (in)efficiency across main online dating stages. *Note*. This figure was produced based on prominent themes in our participants’ narratives.

Our study advances online dating research (e.g., Finkel et al., 2012; Rosenfeld, 2017; Rosenfeld & Thomas, 2012) by focusing on and complicating the notion of efficiency beyond the initial search stage. Adopting a temporal lens (e.g., Harvey, 1989; Lohmeier et al., 2020; Qian & Hu, 2024; Rosa, 2003; Wajcman, 2008), we provide crucial insights into how (in)efficiency unfolds as individuals navigate the online dating process. Based on prominent themes in our participants’ narratives, Figure 1 synthesizes the temporal expectations and experiences, as well as associated expectations and perceptions of (in)efficiency, across the main online dating stages. Drawing on Figure 1, we discuss the three major contributions of our study.

First, our focus on online dating as a multistage process challenges the popular yet simplistic view that extrapolates the benefits of online dating at the search stage to its overall efficiency. This view assumes that gaining easy access to an expanded dating pool at the initial search stage leads to successful relationship formation in a linear, expedited fashion (e.g., Finkel et al., 2012; Qian, 2022; Rosenfeld, 2017; Rosenfeld & Thomas, 2012). As shown in the upper panel of Figure 1, however, online daters often encounter a complex, drawn-out multistage process that typically involves repeatedly initiating online contact, tediously responding to messages, and arduously coordinating the transition from online to offline interactions. Adding to the complexity is that individuals often move back and forth between stages, and the progress may stall or falter at any stage. Thus, rather than entailing a fast and straightforward progression to relationship formation, online dating often involves spending substantial time navigating winding paths, which may well not culminate in forming a relationship. Consequently, many of our participants viewed the process as a waste of time and came to the conclusion that, contrary to their initial expectations, online dating was inefficient. Our findings highlight that research risks overstating the efficiency of online dating should it overlook the lived realities of how online daters navigate the entire process beyond the search stage.

Second, our study foregrounds a temporal lens to understand (in)efficiency and uncovers individuals’ time-related expectations and experiences at different online dating stages to illuminate how digital technologies shape the ways in which individuals “do” dating. As shown in the middle and bottom panels of Figure 1, initially, our participants commonly expected online dating to be time-saving, thereby envisioning the efficiency of online dating through temporal compression. In reality, however, their temporal experiences were far more complex and required greater time investment than expected. In digitally mediated asynchronous communication, men mostly initiated contact and encountered temporal suspension as they waited in limbo for a reply, which led them to question online dating’s efficiency in establishing connections. Women, by contrast, typically received abundant messages and experienced temporal simultaneity. As women often juggled shallow conversations with multiple dating candidates, they came to question online dating’s efficiency in fostering meaningful connections. Transitioning from online to offline interactions engendered significant temporal reconfiguration. Whereas asynchronous online messaging allowed our participants to engage at their own convenience with multiple potential partners simultaneously, offline meetings were typically synchronous, exclusively one-on-one activities that demanded more time to coordinate. After these stages, our participants began to reevaluate and adjust their efficiency expectations. Some decided to take their time in dating and developed a preference for meeting potential partners through offline channels. Taken together, our findings show that the (in)efficiency of online dating is manifested in the complex, dynamic temporal expectations and experiences throughout the process. Moving beyond evaluating the efficiency of online dating through aggregate-level rates of relationship formation, our temporal lens brings to light individual daters’ lived realities to challenge the widely assumed efficiency of online dating. In so doing, our findings offer a new understanding of efficiency from the ground up.

Third, we have uncovered an “efficiency paradox” whereby the promise of efficiency contradicts and amplifies individuals’ lived realities and perceptions of inefficiency in online dating. High efficiency expectations for saving time motivated our participants to opt for online dating in the first place and urged them to move fast within and across online dating stages. Yet, such expectations intensified the sense of inefficiency and frustration when their experiences turned out to be neither fast nor straightforward; rather, what ensued was their far more complex lived realities of temporal suspension, simultaneity, and reconfiguration. The efficiency paradox seems particularly pronounced among minority groups, such as the Chinese immigrants examined in our study. The promised efficiency of online dating is especially appealing to racial minority immigrants as they often face challenges in meeting potential partners through traditional offline venues (Qian, 2022; Schwartz & Velotta, 2018). The Chinese immigrants in our study reported having limited time for socializing, as it can be all-consuming to overcome language and cultural barriers while adapting to study, work, and life in a new country. However, their online dating experiences fell far short of their expectation for temporal compression that online dating would save time. Some of our participants attempted to resolve the efficiency paradox by reevaluating their efficiency expectations and taking their time in dating, which highlights the agency of racial minority immigrants despite the structural challenges facing them in the dating market. Beyond online daters’ individual initiatives, dating platforms could assume more responsibility for addressing the efficiency paradox. For example, dating platforms could offer educational content on the complexities of relationship formation to foster more realistic expectations, make algorithmic adjustments that prioritize compatibility over rapid matching, and develop promotional campaigns that challenge efficiency-oriented dating norms.

Notwithstanding the insights it provides, our study has a few limitations. First, the interviews were conducted in 2018–2019. Since then, the COVID-19 pandemic and new developments in dating technologies may have influenced online daters’ experiences. For example, given a surge of anti-Asian racism and xenophobia since the pandemic (Wu et al., 2021), Chinese immigrants’ experiences of temporal inefficiency may have worsened. Moreover, some platforms have expanded their technological features from supporting online messaging only to also allowing for video-based, synchronous interactions (Duguay et al., 2024). Future research could explore how changing technological features shape new temporalities and (in)efficiency in online dating. Second, while our study only interviewed heterosexual, cisgender Chinese immigrants, future research can explore how temporalities and (in)efficiency of online dating play out in other groups with multiple minority identities, such as queer racial minority immigrants. Finally, we rely on participants’ retrospective accounts rather than longitudinal data to understand the online dating process. Future research could craft a longitudinal design to prospectively capture evolving temporalities throughout the online dating process.

In sum, by foregrounding Chinese immigrant online daters’ temporal expectations, temporal experiences, and their interplays, our study complicates and rethinks the concept of efficiency in online dating. Our findings highlight that digital technologies do not necessarily provide an “efficient” solution to offline challenges. When digital technologies are used to establish a relationship, the pursuit of efficiency, as touted by the dating platforms themselves (e.g., Bumble, 2025; eHarmony, 2024; Plenty of Fish, 2024; Tinder, 2025), may paradoxically exacerbate users’ feelings of inefficiency. This contradiction gives rise to what we term an “efficiency paradox” in online dating, which may be particularly salient in the experiences of minority groups. Building on the theoretical and empirical insights into temporality and efficiency we have developed in this article, we call for more research to critically examine the complexities in everyday practices of digitally mediated intimate lives.
